# Identifying areas of animal welfare concern in different production stages in Danish pig herds using the Danish Animal Welfare Index (DAWIN)

**DOI:** 10.1017/awf.2023.37

**Published:** 2023-06-26

**Authors:** Anne Marie Michelsen, Franziska Hakansson, Vibe Pedersen Lund, Marlene Katharina Kirchner, Nina Dam Otten, Matthew Denwood, Tine Rousing, Hans Houe, Björn Forkman

**Affiliations:** 1 Department of Veterinary and Animal Sciences, University of Copenhagen, Frederiksberg C, Denmark; 2 Department of Animal and Veterinary Sciences, Aarhus University, Tjele, Denmark; 3 Four Paws International, Vienna, Austria

**Keywords:** Animal welfare, assessment protocol, national level, pig, piglet, sow

## Abstract

Animal welfare is of increasing public interest, and the pig industry in particular is subject to much attention. The aim of this study was to identify and compare areas of animal welfare concern for commercial pigs in four different production stages: (1) gestating sows and gilts; (2) lactating sows; (3) piglets; and (4) weaner-to-finisher pigs. One welfare assessment protocol was developed for each stage, comprising of between 20 and 29 animal welfare measures including resource-, management- and animal-based ones. Twenty-one Danish farms were visited once between January 2015 and February 2016 in a cross-sectional design. Experts (n = 26; advisors, scientists and animal welfare controllers) assessed the severity of the outcome measures. This was combined with the on-farm prevalence of each measure and the outcome was used to calculate areas of concern, defined as measures where the median of all farms fell below the value defined as ‘acceptable welfare.’ Between five and seven areas of concern were identified for each production stage. With the exception of carpal lesions in piglets, all areas of concern were resource- and management-based and mainly related to housing, with inadequate available space and the floor type in the resting area being overall concerns across all production stages. This means that animal-based measures were largely unaffected by perceived deficits in resource-based measures. Great variation existed for the majority of measures identified as areas of concern, demonstrating that achieving a high welfare score is possible in the Danish system.

## Introduction

Animal welfare is a concern for consumers and producers and its importance continues to increase (Eurobarometer 2015). Animals at different production stages and ages will encounter differing welfare challenges at varying points of their development. When assessing the overall welfare of a production animal, it is important therefore to assess each stage separately. Equally, in seeking to improve welfare, it is important to know at which point in an animal’s lifespan is its welfare most impaired. While there are clearly welfare challenges associated with pig production, there has yet to be a comprehensive attempt to identify the importance of each challenge (calculated as the welfare impact on the individual animal multiplied by the prevalence) for each age group. If money is to be allocated to welfare improvements, it is therefore vital to become acquainted with the welfare impact of specific challenges, not to mention the economic cost of any proposed changes. The current study focuses on the first of these two requirements. While existing schemes such as Welfare Quality® (WQ) have been developed to measure animal welfare at farm level, they are not suited for the present task. There is still no official aggregation procedure for sows or piglets, and both sows and piglets are measured as ‘one unit.’ Another reason is that because the emphasis of WQ is on welfare status at farm or group level, the aggregation procedures have been adjusted for this purpose, making the protocols less suitable for assessing the importance of each specific welfare challenge (for a longer discussion, see de Graaf *et al.* 2017, 2018; Sandøe *et al.* 2017). For these reasons, new animal welfare protocols have been developed which are more suited to the current purpose, as well as being specifically applicable to Danish conditions. The protocols were designed to collect data for national Danish Animal Welfare Indices (DAWIN) (see also Otten *et al.* 2020). The purpose of the indices is to provide an overview of the animal welfare status on a national level and to make it possible for changes in animal welfare to be monitored over time – both in terms of overall welfare and changes in the prevalence of specific welfare problems in pig production. The objective of this study was to identify areas of animal welfare concern in different production stages in Danish pig herds using the DAWIN protocols.

## Materials and methods

### Development of animal welfare assessment protocols

DAWIN protocols were developed for each of the following four animal groups: (1) gestating sows and gilts; (2) lactating sows; (3) piglets; and (4) weaner-to-finisher pigs. The DAWIN protocols were based on the four welfare principles used in the WQ protocols: Good feeding, Good housing, Good health and Appropriate behaviour (Keeling 2009). In a preliminary step, four lists of potential measures were developed based on existing animal welfare assessment protocols and a literature review, with additional input from a workshop involving national and international experts. The selection of measures for the final DAWIN protocols was based on a literature review assessing the validity, repeatability and feasibility of each measure along with on-farm feasibility testing for new or modified measures. Existing measures were taken from the WQ pig protocol (Welfare Quality® 2009), although in some cases, levels were slightly modified or the number reduced to enhance feasibility, as for example, the measure ‘Body condition score’ for lactating sows and gestating sows and gilts, where three levels in the WQ protocol (0, 1, 2) were merged into two in the DAWIN protocol (0, 1). The final DAWIN protocols for gestating sows and gilts, lactating sows, piglets and weaner-to-finisher pigs include 29, 26, 20 and 23 measures, respectively, including animal-, resource- and management-based measures (Table 1; see also Tables I to IV in Supplementary material for a short version of the original protocol). Data for the animal-based measure ‘Liver disease’ were provided by the Danish Veterinary and Food Administration (DVFA). The number of levels indicating differing severities of a measure ranged from two (presence/absence) and three (absence, moderate presence and severe presence) to five (categorical nuances).

**Table 1.** Welfare principles (as defined by WQ) and recorded measures for gestating sows and gilts, lactating sows, piglets and weaner-to-finisher pigs classified into animal- (AB), resource- (RB) and management-based (MB) measures

Welfare principle	Type of measure	Measure			
		Gestating sows and gilts	Lactating sows	Piglets	Weaner-to-finisher pigs
Good feeding	AB	Body condition score	Body condition score	–	Body condition score
	RB	Roughage, Feeding system^#^, Water supply, Water cleanliness	Roughage, Feeding system^#^ **, Water supply, Water cleanliness	Teats per piglet, Water supply, Water cleanliness	Feeding system, Water supply, Water cleanliness
	MB	–	–	Age at weaning	–
Good housing	AB	Slipperiness of the floor, Manure on the body, Bursitis, Panting	Manure on the body, Bursitis, Panting	Manure on the body	Manure on the body, Slipperiness of the floor, Panting
	RB	Type of housing – service unit, Type of housing – gestation unit, Stocking density^#^, Resting area – Floorage^#^, Resting area – Floor type^+^, Crate space – Service unit, Cooling system^#^	Farrowing system, Farrowing rails, Space in farrowing system^¤^, Resting area – Floorage^#^ **, Resting area – Floor type^¤^	Stocking density, Resting area – Floorage, Resting area – Floor type, Access to teats	Resting area – Floorage, Resting area – Floor type, Cooling system, Stocking density
	MB	Duration of crating – service unit	Long-term crated sows	–	–
Good health	AB	Hampered respiration, Shoulder wounds, Lameness, Integument alterations, Prolapse, Vulvar lesions, Hernia, Overgrown claws, Nose ring	Hampered respiration, Shoulder wounds, Integument alterations, Vulvar lesions, Prolapse, Hernia, Nose ring, Overgrown claws	Hampered respiration, Lameness, Lesions on the body, Carpal lesions, Neurological symptoms, Diarrhoea, Rectal prolapse, Splay legs	Hampered respiration, Lameness, Integument alterations, Tail-damage, Ear-damage, Rectal prolapse, Hernia, Twisted snout, Neurological symptoms, Liver disease*
	RB	Hospital pens	Hospital pens	–	Hospital pens
	MB	–	–	Castration, Tail docking, Ear notching	–
Appropriate behaviour	AB	Stereotypies	Stereotypies	–	–
	RB	Rooting material	Rooting material	–	Rooting material
	MB	–	Nest building	–	–

# Measure only applies to group-housed sows.

* Measure derived from database.

** Not applicable due to the housing systems present in the sample.

+ Integrated measure related to group-housed and crated sows.

¤ Applies to crated sows and sows housed in groups and individual pens.

### Study population and design

The study was designed to be cross-sectional where farms were visited once between January 2015 and February 2016. Farm recruitment was initially based on stratified random sampling with regard to system (grower-to-finisher farms, sow-to-finisher farms and sow farms with and without weaned pigs) and sow and slaughter pig mortality. In addition, an inclusion criterion of a minimum of 200 sows or 500 slaughter pigs was used. In the attempt to recruit 90 farms, a total of 447 were initially contacted via letter or telephone and asked to participate. Participation in the project was voluntary. Overall, 17% of the selected farms agreed to participate which meant additional farms had to be recruited using convenience sampling, resulting in a total of 81 commercial conventional Danish farms. In the convenience sampling, attempts were made to obtain a certain number of farms for the different systems, but it was not possible to stratify the selection of farms based on mortality. For the 81 farms, 25 were slaughter pig farms (grower-to-finisher farms), 26 were sow farms without weaned pigs and 30 were sow-to-finisher or sow farms with weaned pigs. Data were collected by two universities, but due to a misunderstanding two slightly different protocols were used affecting several measures. As agreement in the total data set could not be ensured, it was decided to use the most standardised and consistent data set, resulting in a subsample of 21 farms. These 21 farms were all visited by assessors from one institution. Of these, 19 were sow-to-finisher farms or sow farms with weaned pigs and two were sow farms without weaned pigs. The subsample is described further in the *Results.*

### Observer training

Five observers performed the assessments in the 21 farms and, where ever possible, the same observer collected data from a particular animal group. Prior to the study period, all observers participated in an official WQ training course followed by further practical training for the measures specific to the DAWIN. Additional observer training was conducted after six months of data collection. No inter-observer reliability testing was performed.

### Sampling of animals and data collection

Farm visits started with a tour of the facility, where a sketch was made of pens and sections within each barn. This sketch formed the basis for stratified random sampling of pens that took into account the different barns, sections, location of pens within sections, numbers of animals in each section and age groups or stages of gestation and lactation. Animals from pens where mixing or vaccination had taken place during the week prior to the visit were not included in the sample, unless dynamic groups were practiced, i.e. where animals are continuously added to the group. Animals kept in hospital pens at the time of the visit were not included in the sample. Sample sizes for each animal group were based on the WQ protocols and were as follows: 30 gestating sows and gilts (ten from the service unit and 20 from the gestation unit), ten lactating sows plus their litters, and 150 weaner-to-finisher pigs. Weaner-to-finisher pigs were sampled from ten pens, and 15 pigs from each pen were assessed. In some cases fewer than 15 pigs were present in a pen and the remaining pigs were sampled from the pen to the right. For sows and gilts, the number of sows assessed in each pen depended on the total number in the pen, i.e. a proportion of animals in the selected pens were assessed. A larger proportion of animals were selected from pens with fewer animals compared to pens with a large number of animals. For group-housed animals, sampling within pens was performed as described in the WQ protocol (Welfare Quality® 2009). Data were collected using the DAWIN protocols corresponding to the animal groups present on the farm. For all four animal groups, resource-based measures were assessed at pen level whenever animals from that pen were included in the sample. All hospital pens for each group were assessed, regardless of the presence of animals in the pen. However, water supply and floor type in hospital pens were only assessed for the pens in use. With the exception of panting and stereotypies, all animal-based measures were assessed on an individual level by direct observation within a short distance of the sampled animals. Panting was assessed from a distance without disturbing the animal, but still within a range of visibility from inside the pen or prior to entering the pen. For lactating sows and gestating sows and gilts, panting was assessed at individual animal level, while for weaner-to-finisher pigs it was at pen level. Similarly, stereotypies were assessed at animal level from a distance. Animal-based clinical measures were assessed on one or both sides of the animal depending on the measure (see Tables I to IV in Supplementary material). Management-based measures were collected at farm level by interviewing the farmer during the farm visit. For gestating sows and gilts, the measures ‘Feeding system’, ‘Stocking density’, ‘Resting area – Floorage’ and ‘Cooling system’ were only applicable for group-housed animals, while ‘Resting area – Floor type’ was assessed depending on the housing system, i.e. crated, individually penned (not present in our sample) or group-housed. Likewise, some measures for lactating sows were only applicable to either crated or group-housed animals, while others differed depending on the housing system. However, only farms with crated lactating sows were included in the sample and measures only applicable to group-housed lactating sows, i.e. ‘Feeding system’ and ‘Resting area – Floorage’ will therefore not be considered further.

### Data processing

Data were transcribed from paper records into electronic format by the three main observers that had recorded the data on-farm. These electronic records were then processed using a series of bespoke functions written in the R statistical programming language (R Core Team 2017) in order to produce a single score corresponding to each combination of farm and previously defined levels in the DAWIN protocols (Tables I to IV in Supplementary material). During this process, the data were also quality checked against a series of pre-specified expectations (maximum/minimum values for numeric data, pre-defined permissible categories for categorical data) and any errors detected in the data were checked and manually corrected before re-running the code.

### Aggregation procedure and descriptive statistics

An expert panel was used to assess the welfare value of each measure. The panel was recruited by contacting three different groups who were asked to appoint their experts: the Danish Veterinary Association; SEGES (the main agricultural organisation in Denmark); and the section for assessing animal welfare in the DVFA. The aim was to have ten experts from each group. The scientists involved in the current project were also asked to complete the questionnaire. From the 29 experts and scientists who received the questionnaire, 26 answered (four consultants from SEGES, 16 animal welfare controllers from the DVFA and six scientists). However, not all experts answered all questions.

The experts were asked to answer questions about each possible outcome for a measure, e.g. ‘What is the welfare of a pig with 10 to 30% of its body covered in faeces?’ and ‘What is the welfare of a pig with more than 30% of its body covered in faeces?’ The score should be a number between 0 (worst possible welfare) and 100 (best possible welfare), where 50 or above was defined as acceptable welfare, a score below 50 indicated that something should be done about the problem, and 20 or below was considered as unacceptable welfare. This was inspired by the WQ approach. The assigned expert score for each level of a measure was calculated as the mean value of all expert scores (see Table I to IV in Supplementary material for measures of variability of expert scores). For a given measure recorded at animal level, the on-farm prevalence of each outcome was multiplied by the assigned expert score.

To take an example: according to the protocol for gestating sows and gilts, the mean expert score for the welfare of a sow with 10 to 30% of its body covered in faeces was 45, and for those with more than 30% covered in faeces it was 30. For one of our sample farms, the corresponding proportion of pigs with less faecal contamination was 0.43, and the proportion of pigs with more faecal contamination was 0.2. The farm measure score for ‘Manure on the body’ was therefore 100 × 0.37 + 45 × 0.43 + 30 × 0.2 = 62. To derive the measure score for ‘Manure on the body’ of 64.7, the median of all 21 farms was calculated. To adjust for varying numbers of weaner-to-finisher pigs in the pens, a weighted average per pen was used to calculate each farm measure score for this animal group.

The resulting measure scores are presented as median, minimum, maximum and interquartile range and given for each animal group and for each measure. Additionally, the number of farms included for each measure and the percentage of farms with a farm measure score below 50 (a score of 50 or above is considered as acceptable welfare according to the definition given in the expert questionnaire) is given. Divided into animal- and resource-based measures, farm welfare scores calculated as mean of all measures for each animal group and overall farm welfare scores calculated as mean of all animal groups within a farm are presented. Management-based measures were included as either animal- (‘Age at weaning’, ‘Castration’, ‘Tail docking’ and ‘Ear notching’) or resource-based measures (‘Duration of crating – service unit’ and ‘Long-term crated sows’). Any missing values for given measures were replaced by the population mean to achieve complete farm scores.

### Classification as area of animal welfare concern

A given measure is classified as an area of animal welfare concern whenever the median measure score of all farms is below 50, which in all cases mean that more than 50% of the farms have a farm measure score below the threshold for acceptable welfare.

## Results

### Herd demographics

The number of sows in the 21 farms ranged from 200 to 1,400 with a mean of 541 sows. In comparison, the mean number of sows in Danish sow-to-finisher farms and sow farms with weaned pigs was 451 and 855, respectively, in 2015 (Statistic Denmark 2015). In the current study, the housing system for gestating sows and gilts differed slightly between included farms. In the service unit, sows were either crated or group-housed, while all sows in the gestation unit were group-housed due to EU legislative requirements (European Union Directive 2019). On all farms, weaner-to-finisher pigs and lactating sows were group-housed and crated, respectively. These housing systems resemble the systems commonly used on Danish pig farms. Farms were located all over Denmark with the exception of Northern Jutland.

### Gestating sows and gilts

Resulting measure scores for gestating sows and gilts are presented in Table 2. Median measure scores ranged from 32.6 to 100, with six measures (‘Type of housing – Service unit’, ‘Duration of crating – Service unit’, ‘Resting area – Floorage’, ‘Water supply’, ‘Resting area – Floor type’ and ‘Roughage’) scoring below 50 (classified as areas of animal welfare concern). The percentage of farms with a measure score below 50 (threshold for acceptable welfare) ranged from 52 to 95% for the six measures.

**Table 2.** Measure scores for gestating sows and gilts from 21 Danish pig herds presented as median, minimum, maximum, interquartile range, total number of farms and number and percentage of farms scoring below 50 (cut-off point for ‘acceptable welfare’). Measure scores are ranked by median

	Measure score					
Measure	Median	Min	Max	IQR	N	N (%) below 50
Type of housing – Service unit	32.6	32.6	58.5	32.6 - 32.6	19	17 (90)
Duration of crating – Service unit	33.8	23.3	100	33.8 – 33.8	21	20 (95)
Resting area – Floorage	35.2	29.2	100	29.2 – 43.8	20	17 (80)
Water supply	39	20	100	24.9 – 95.1	21	11 (52)
Resting area – Floor type	45.9	35.6	98.7	45.8 – 46.0	21	19 (90)
Roughage	46.5	33.4	100	33.4 – 83.5	21	11 (52)
Crate space – Service unit	50.6	17.6	83.5	34.5 – 56.7	18	8 (44)
Feeding system	53.6	28.9	100	30.2 – 94.4	18	8 (44)
Manure on the body	64.7	37.7	79.7	59.1 – 73.5	21	3 (14)
Hospital pens	73.5	36.5	100	72.2 – 84.5	21	3 (14)
Stereotypies	73.5	50.2	100	67.6 – 89.1	21	0
Bursitis	75.7	34.4	100	49.0 – 92.7	21	7 (33)
Stocking density	79.5	29.3	100	56.6 – 96.6	21	5 (24)
Rooting material	80.8	20	100	31.9 – 98.8	21	7 (33)
Integument alterations	87.6	68.8	100	80.2 – 93.2	21	0
Slipperiness of the floor	90.1	52.6	100	85.7 – 96.5	21	0
Overgrown claws	90.7	52.8	100	87.2 – 97.4	21	0
Lameness	92.2	63.2	100	88.4 – 100	21	0
Vulvar lesions	96.3	70.3	100	94.4 – 98.1	21	0
Water cleanliness	97.1	37.6	100	80.2 – 100	21	2 (10)
Cooling system	98.2	31.3	100	35.9 – 100	21	8 (38)
Body condition score	100	88.3	100	98.3 – 100	21	0
Shoulder wounds	100	93.7	100	97.4 – 100	21	0
Type of housing – Gestation unit	100	82.7	100	100 – 100	21	0
Hampered respiration	100	96.1	100	100 – 100	20	0
Prolapse	100	97.1	100	100 – 100	21	0
Hernia	100	100	100	100 – 100	21	0
Nose ring	100	100	100	100 – 100	21	0
Panting	100	100	100	100 – 100	15	0

The six measures identified as areas of animal welfare concern were all resource-based. For the type of housing in the service unit, loose-housing (group housing or individual pens) was considered to be the best possible animal welfare alternative. However, none of the farms within the sample had all of the sows in the service unit loose-housed at the time of the visit. Duration of crating in the service unit (based on information from the farmer) varied between farms from no crating to crating for more than four weeks. In terms of ‘Resting area – Floorage’ (resting area was defined as the area with the best possible floor type), the best possible welfare was considered to be when all sows were able to lie in half recumbence in the resting area at the same time. This corresponded to an area of 1.3 m^2^ for a sow of 250 kg (0.033 × live weight^0.66^; Petherick & Baxter 1981). Results, however, showed that the resting area was on average 1.0 m^2^ per sow, with four farms providing less than 0.5 m^2^ per sow. The primary floor type of the resting area for group-housed sows was solid concrete flooring. Likewise, the floor type of the lying area of crated sows in the service unit was exclusively solid concrete floor under the front end of the sows and slatted floor under their rears.

### Lactating sows

Resulting measure scores for lactating sows are presented in Table 3. Median scores ranged from 23.1 to 100, with five measures (‘Nest building’, ‘Space in farrowing system – Crate’, ‘Roughage’, ‘Farrowing system’ and ‘Resting area – Floor type – Crate’) scoring below 50. All farms were given the minimum score for ‘Nest building’ and ‘Farrowing system’ (23.1 and 33.8, respectively), resulting in 100% of the farms scoring below 50. Similarly, none of the farms had a farm measure score above 50 for ‘Resting area – Floor type – Crate.’ For the two measures ‘Space in farrowing system – Crate’ and ‘Roughage’, 62 and 95% of the farms scored below 50, respectively.

**Table 3.** Measure scores for lactating sows from 21 Danish pig herds presented as median, minimum, maximum, interquartile range, total number of farms and number and percentage of farms scoring below 50 (cut-off point for ‘acceptable welfare’). Measure scores are ranked by median

	Measure score					
Measure	Median	Min	Max	IQR	N	N (%) below 50
Nest building	23.1	23.1	23.1	23.1 – 23.1	21	21 (100)
Space in farrowing system – Crate	33.3	16.7	75	25.0 – 50.0	21	13 (62)
Roughage	33.4	33.4	100	33.4 – 33.4	21	20 (95)
Farrowing system	33.8	33.8	33.8	33.8 – 33.8	21	21 (100)
Resting area – Floor type – Crate	47.5	29.4	47.5	47.5 – 47.5	21	21 (100)
Bursitis	63.6	43.3	100	59.5 – 72.7	21	2 (10)
Rooting material	69.7	20	100	52.0 – 90.5	20	4 (20)
Hospital pens	73.5	36.5	100	72.2 – 84.5	21	3 (14)
Manure on the body	77.8	63.8	100	74.9 – 83.5	21	0
Overgrown claws	91.4	46	100	80.7 – 100	21	1 (5)
Stereotypies	92.2	53.1	100	88.8 – 100	20	0
Long-term crated sows	94.8	52.3	100	87.6 – 96.0	18	0
Farrowing rails	100	25.3	100	25.3 – 100	21	6 (29)
Vulvar lesions	100	70.3	100	86.5 – 100	21	0
Integument alterations	100	85.1	100	92.6 – 100	21	0
Shoulder wounds	100	90	100	92.2 – 100	21	0
Water cleanliness	100	80.2	100	100 – 100	21	0
Prolapse	100	91.4	100	100 – 100	21	0
Panting	100	91.8	100	100 – 100	21	0
Water supply	100	92	100	100 – 100	21	0
Body condition score	100	100	100	100 – 100	21	0
Hampered respiration	100	100	100	100 – 100	21	0
Hernia	100	100	100	100 – 100	21	0
Nose ring	100	100	100	100 – 100	21	0

As with gestating sows and gilts, all measures detected as areas of concern were related to resources. For ‘Nest building’ all farms were given the lowest possible score, regardless of the presence of nest-building material. According to the DAWIN protocol, sows should be in loose-housing to be able to display nest-building behaviour, and none of the farms included had loose-housed sows in the farrowing unit. Likewise, the lowest score was given for ‘Farrowing system’ due to all sows being crated in the farrowing unit. For the measure ‘Roughage’, only one farm was given the maximum score, while the remaining farms scored below 50. None of the farms achieved the maximum score for the measure ‘Space in farrowing system – Crate’, meaning that space requirements were not fulfilled for all sows on any of the farms. The primary floor type of the resting area in the farrowing unit was solid concrete flooring under the front of the sow and slatted elsewhere, which was the case in 20 out of 21 farms.

### Piglets

Resulting measure scores for the piglets are presented in Table 4. Median scores ranged from 28 to 100, with seven measures (‘Ear notching’, ‘Resting area – Floorage’, ‘Tail docking’, ‘Access to teats’, ‘Castration’, ‘Carpal lesions’ and ‘Resting area – Floor type’) scoring below 50. In addition, 71 to 100% of the farms scored below 50 for the seven measures. For the measure ‘Access to teats’, all farms were given the minimum score (36.1), while the greatest range was seen for the measure ‘Castration’ (14.3 to 100).

**Table 4.** Measure scores for piglets from 21 Danish pig herds presented as median, minimum, maximum, interquartile range, total number of farms and number and percentage of farms scoring below 50 (cut-off point for ‘acceptable welfare’). Measure scores are ranked by median

	Measure score					
Measure	Median	Min	Max	IQR	N	N (%) below 50
Ear notching	28	28	100	28.0 – 100	21	15 (71)
Resting area – Floorage	29	25.2	33.5	27.0 – 30.9	20	20 (100)
Tail docking	30.6	21.4	30.6	21.4 – 30.6	21	21 (100)
Access to teats	36.1	36.1	36.1	36.1 – 36.1	21	21 (100)
Castration	37	14.3	100	37.0 – 37.0	21	20 (95)
Carpal lesions	37.7	25.5	59.8	28.4 – 41.3	21	20 (95)
Resting area – Floor type	41.2	41.2	71.7	41.2 – 44.7	21	17 (81)
Stocking density	73.1	53.7	100	64.4 – 93.2	21	0
Diarrhoea	90.3	61.3	100	84.7 – 93.6	21	0
Teats per piglet	91.3	69	100	89.8 – 100	21	0
Lameness	91.9	67	100	83.7 – 100	21	0
Lesions on the body	92.6	67	100	90.8 – 100	21	0
Manure on the body	96.1	66	100	92.9 – 100	21	0
Age at weaning	100	46	100	100 – 100	21	4 (19)
Water cleanliness	100	91.9	100	100 – 100	21	0
Neurological symptoms	100	90.2	100	100 – 100	21	0
Hampered respiration	100	91.2	100	100 – 100	21	0
Rectal prolapse	100	100	100	100 – 100	21	0
Splay leg	100	100	100	100 – 100	21	0
Water supply	100	100	100	100 – 100	21	0

For piglets, a total of seven measures were detected as areas of concern. All but one (‘Carpal lesions’) of the detected areas were management- and resource-based and related to housing and farm management routines. None of the farms fulfilled the requirements to be classified as acceptable in terms of animal welfare for the measures ‘Tail docking’, ‘Resting area – Floorage’ (determined by the covered creep area with adjacent solid floor) and ‘Access to teats.’ For ‘Resting area – Floor type’, four out of 21 farms scored above the acceptable threshold. The main floor type in the resting area was concrete, in some cases covered with a hard rubber mat and/or sparse bedding material. Bedding was provided on some farms, especially for the younger piglets. Tail docking was performed on all 21 farms, but 15 of these used analgesics for male piglets as tail docking occurred at the same time as castration. Similarly, castration was performed on 20 out of 21 farms, 19 of which used analgesics. Ear notching was performed on 15 out of 21 farms, while on the remaining farms ear-tag numbers were used for identification.

### Weaner-to-finisher pigs

Resulting measure scores for the weaner-to-finisher pigs can be found in Table 5. Median scores ranged from 25.3 to 100, with six measures (‘Water supply’, ‘Cooling system’, ‘Resting area – Floorage’, ‘Rooting material’, ‘Resting area – Floor type’ and ‘Hospital pens’) scoring below 50. The greatest variation between farms was seen for the measure ‘Hospital pens’ with a minimum score of 13.7 and a maximum of 100. For the six measures, 53 to 95% of the farms had a farm measure score below 50.

**Table 5.** Measure scores for weaner-to-finisher pigs from 19 Danish pig herds presented as median, minimum, maximum, interquartile range, total number of farms and number and percentage of farms scoring below 50 (cut-off point for ‘acceptable welfare’). Measure scores are ranked by median

	Measure score					
Measure	Median	Min	Max	IQR	N	N (%) below 50
Water supply	25.3	20.5	91.7	20.5 – 40.4	19	15 (79)
Cooling system	29.0	29.0	100	29.0 - 100	19	12 (63)
Resting area – Floorage	32.5	27.7	70	27.7 – 51.8	19	13 (68)
Rooting material	46.6	29.8	100	41.0 – 85.7	19	10 (53)
Resting area – Floor type	47.2	35.2	89.6	46.4 – 47.2	19	18 (95)
Hospital pens	47.2	13.7	100	21.3 – 100	19	11 (58)
Stocking density	63.3	27.5	100	51.3 – 81.5	19	5 (26)
Slipperiness of the floor	78	44.9	100	68.2 – 85.7	19	1 (5)
Ear-damage	81.7	64.9	100	76.8 – 87.2	19	0
Manure on the body	89.6	73.1	98.4	84.6 – 93.7	19	0
Water cleanliness	92.2	52.5	100	82.3 – 100	19	0
Tail-damage	94.9	72.7	100	89.5 – 99.3	19	0
Integument alterations	97.4	80.8	100	95.6 – 98.4	19	0
Lameness	97.8	90.3	100	97.2 – 98.6	19	0
Body condition score	98.6	91	100	97.4 – 99.2	19	0
Hernia	99.5	96.5	100	98.9 – 99.9	19	0
Neurological symptoms	100	97.9	100	99.4 – 100	19	0
Rectal prolapse	100	99.2	100	99.4 – 100	19	0
Hampered respiration	100	99.5	100	100 – 100	19	0
Twisted snout	100	99.4	100	100 – 100	19	0
Panting	100	99.7	100	100 – 100	19	0
Liver disease	100	100	100	100 – 100	12	0
Feeding system	100	100	100	100 – 100	19	0

The six measures identified as areas of concern were all resource-based and related to feeding (‘Water supply’) and housing (‘Resting area – Floorage’, ‘Resting area – Floor type’, ‘Cooling system’, ‘Hospital pens’ and ‘Rooting material’). For the measure ‘Water supply’, ten out of 19 farms had, on average, more than 15 pigs per nipple, and five farms provided only one nipple per pen. A cooling system consisting of sprinklers above the pens was present at 14 out of 19 farms. Only one farm in the sample provided deep bedding in most of the pens assessed and therefore achieved a high measure score for ‘Resting area – Floor type.’ The remaining 18 farms scored below 50, with 13 farms providing a solid resting area and five providing slatted or drained flooring (where openings constitute a maximum of 10% of the area) in some or all pens. For ‘Resting area – Floorage’ (resting area was defined as the area with the best possible floor type), all pigs present in a pen should be able to lie in half recumbence, corresponding to an area of 0.31 m^2^ per pig of 30 kg body weight (0.033 × live weight^0.66^; Petherick & Baxter 1981). However, our results showed that, on average, the resting area provided for a pig of 30 kg was 0.22 m^2^. Sufficient rooting material was present on nine out of 19 farms. Two farms had no hospital pen in the weaner or finisher pig unit, and six of the remaining farms did not have space (according to the requirements listed in the DAWIN protocol) for an additional sick or injured animal within their hospital pens at the time of the visit. One farm provided deep bedding for the resting area of the hospital pens, one farm had a slatted floor, and the remaining 15 farms had solid concrete floor or hard mats.

### Farm welfare score

**Figure 1.**
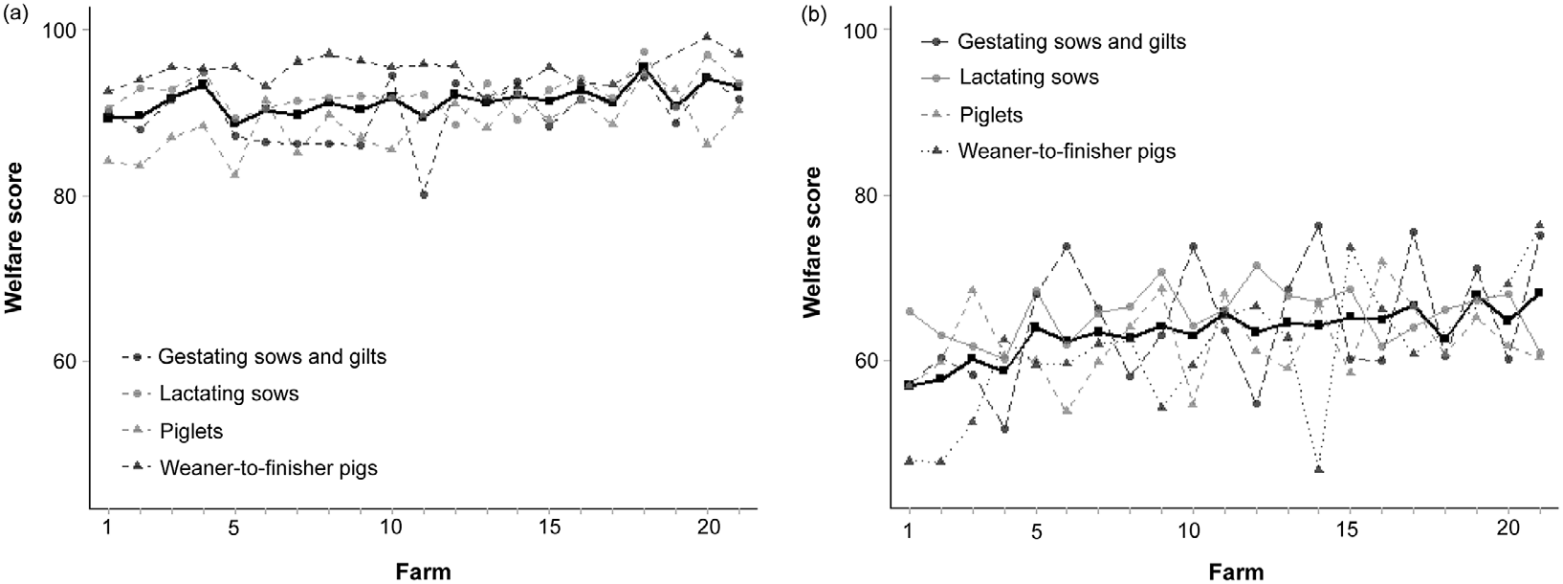
Farm welfare scores for each animal group and overall farm welfare scores (solid black line) for (a) animal-based measures and (b) resource-based measures for the 21 farms. Farms are listed by increasing total farm welfare score.

Farm welfare scores (mean of all measures within a farm) for each of the four animal groups divided into animal- and resource-based measures are displayed in Figure 1. The farms are listed in order of increasing total farm welfare score (animal-based + resource-based measures). When comparing the animal- and resource-based measures a higher score was generally seen for animal-based measures where the highest was achieved for weaner-to-finisher pigs. Moreover, greater variation between farms for each animal group and between animal groups within farms was seen for resource-based measures. For animal-based measures the mean of all animal groups (black line) was largely unchanged (around 90) from the farm with the lowest to the farm with the highest total farm welfare score, while for resource-based measures the mean was increased by about ten (from 60 to 70).

## Discussion

This study uncovered several areas of welfare concern at different stages of pig production. A total of 24 measures were classified as areas of welfare concern (defined as a median measure score below 50), and many of the measures were related to housing for the four groups of pigs, suggesting a more general problem relating to housing facilities in Danish herds. When comparing the welfare at different stages of the production cycle, the results highlight inadequate available space as a general concern, with another being the type of floor in the resting area. Only one of the animal-based measures was classified as an area of animal welfare concern namely carpal lesions for piglets.

The greatest number of measures with negative welfare consequences was detected for piglets, with tail docking, size of the resting area and access to teats being areas of concern on all the visited farms. On average, fewer than 50% of the piglets in the assessed pens were able to lie in full lateral recumbence in the resting area at the time of weaning. The area required was calculated using the formula given by Petherick and Baxter (1981; but see also Fels *et al.* 2016). Here, the main floor type in the resting area was concrete, which was not considered to be acceptable for the welfare of piglets by the experts. The inadequate flooring was also reflected by the frequently found carpal lesions of the piglets. This is in agreement with other studies which have shown concrete flooring in resting and suckling areas to be associated with a greater prevalence of carpal lesions compared to other kinds of bedding (Ziron & Hoy 2003; Zoric *et al.* 2009). However, bedding was provided on some farms, especially for the younger piglets, leading to a higher welfare score.

Piglets’ access to the sows’ teats was given the lowest possible score on all farms due to insufficient space availability when suckling. On most farms, space was only insufficient on one side of the crate. The space requirements were based on the average length of a piglet at four weeks of age (Moustsen & Poulsen 2004) and it is therefore likely that access to teats, and consequently growth (Moustsen & Duus 2006a), was mainly compromised late in the suckling period.

Three of the seven measures detected as areas of concern for piglets were related to management-induced pain. Tail docking was performed on all of the farms, which resulted in the low score for this measure. A large proportion of the farms used analgesics for male piglets as tail docking and castration were performed in succession. According to Danish legislation, analgesics must be administered for castration of piglets (Ministry of Food, Agriculture and Fisheries 2020). Analgesics were used for castration on all but one farm. The efficacy of analgesics for castration and tail docking is disputed (e.g. Nannoni *et al.* 2014), and the experts still considered both tail docking and castration to impair animal welfare despite the use of analgesics. In addition to ear notching, which was performed on the majority of farms, ear-tags were used for identification of selected piglets. Ear-tagging was selected in the expert questionnaire as being the reference standard and therefore set to 100 (best possible welfare). However, Leslie *et al.* (2010) compared the pain response for the two procedures and did not find considerable differences. It is therefore possible that marking for identification is an even greater area of concern than reported here.

Housing systems where sows are kept in crates were not considered acceptable by the experts. In addition, both crate size (for lactating sows) and duration of crating (in the service unit) were considered to decrease the welfare of individual animals. For crates to be deemed adequate in size, dynamic space requirements had to be met for lactating sows. According to Moustsen and Duus (2006b), these space requirements will allow sows to stand up and lay down unhindered which is in accordance with the national legislation (Ministry of Food, Agriculture and Fisheries 2020). However, when executed by the authorities, space assessments are much less strict and only require crates to be at least equal to the length of the sow (Ministry of Food, Agriculture and Fisheries 2022). The high prevalence of crates that the DAWIN protocol deemed ‘not suitable’ may be indicative of the variation that exists between the ideal as expressed in legislation and the control as it is carried out. The use of crates was related to another area of concern – the prevention of nesting behaviour by sows in the farrowing section. It has been shown that farrowing sows have a high motivation to perform nest-building behaviour (Arey 1992). Although nest-building material was provided to some extent on most farms, all were given the lowest possible score based on the fact that all sows were crated in the farrowing unit and the effect of providing nest-building material to crated sows is limited (Thodberg *et al.* 2002).

As for the piglets, the floor type of the resting area was also detected as an area of concern for the remaining animal groups. The European Union Directive (2019) states that the lying area must be ‘physically and thermally comfortable.’ We do not believe that this was met for our sample, where the majority of farms had solid concrete flooring in the resting area. In addition, the floorage of the resting area was inadequate for group-housed animals on the majority of farms.

For weaner-to-finisher pigs, the measure ‘Water supply’ had the lowest median score. In the DAWIN protocol the threshold for sufficient water supply was set to ten pigs per nipple, which is the recommendation of the Royal Society for the Prevention of Cruelty to Animals (RSPCA 2016) and the threshold used in the WQ protocol. However, the recommendation from the main Danish farmers’ organisation is up to 15 pigs per nipple (SEGES 2016) and the prevalence of the perceived problem was therefore not entirely unexpected. However, on more than half of the farms, the number of pigs per nipple surpassed even this recommendation. While British animal welfare standards use a threshold of ten pigs per nipple for feed-restricted pigs and 15 for unrestricted pigs (DEFRA 2020), we wish to emphasise that there is little scientific literature either supporting or disputing the experts’ evaluation that more than ten or 15 pigs per nipple will lead to serious welfare consequences. The same considerations apply for gestating sows and gilts, where water supply was also identified as an area of concern. Although detected as an area of concern for gestating sows and gilts and weaner-to-finisher pigs, the measure ‘Water supply’ received median scores of 100 for lactating sows and piglets. It is noteworthy that water supply has also emerged as an important animal welfare issue in other protocols and species (e.g. Meyer-Hamme *et al.* 2018; Otten *et al.* 2020)

The measure ‘Cooling system’ was identified as an area of concern for weaner-to-finisher pigs. To comply with current Danish legislation, a means of cooling must be available for all pigs above 20 kg (Ministry of Food, Agriculture and Fisheries 2020). In the assessed farms, sprinklers were the only cooling system identified (located over slatted or drained flooring). However, our farm visits and the individual cooling routines often did not coincide and, for feasibility reasons, the availability but not the functionality of the sprinklers was assessed. Therefore, this assessment was to some extent subjective and may not be a true reflection of on-farm usage.

Within this study, the availability of a pen to separate injured or sick animals from the group was weighted by the experts as highly important for the welfare of an individual animal, which also corresponds with Danish legislation (Ministry of Food, Agriculture and Fisheries 2022). However, on more than half of the farms with weaner-to-finisher pigs, the availability of hospital pens was limited or the pens were not sufficiently equipped in terms of suitable bedding or water supply.

Limited access to roughage was an area of concern for lactating sows especially, but also for gestating sows and gilts. The feeding regimes of sows, with highly concentrated feed given a few times per day, along with restricted feeding during the gestation period, have the potential to negatively affect sows by inducing hunger and frustration (Meunier-Salaün *et al.* 2001). For weaner-to-finisher pigs, the availability and suitability of rooting material were an area of concern. When rooting material was provided it was often deemed to be of an insufficient quality according to the experts’ assessments (see also Studnitz *et al.* 2007).

It is worth noting that although there were a relatively large number of areas of concern based on the resource- and management-based measures, very few of these resulted in consequences for the animal-based measures. The number of resource-based measures approximately matched the number of animal-based measures in the protocols. One possible explanation for the numerous resource- and management-based measures might be the mismatch between the experts’ perception of acceptable animal welfare, and the current legal requirements, which serve as guidelines for the farmers. For example, while crating of sows might be considered inadequate as regards sow welfare, it is legal by Danish legislation (Ministry of Food, Agriculture and Fisheries 2020). Another possible explanation for the observed difference may be that the thresholds for the resource-based measures were set lower compared to those for animal-based measures. However, the process and sources of background information used to set the thresholds were the same for both types of measures. For ease of use the protocols have few levels for each measure and this might have affected whether or not a certain measure registered as an area of concern. For example, for the measure lameness in gestating sows and gilts, an animal was only scored as lame when ‘minimal weight-bearing on the affected limb’ or ‘inability to walk’ were observed, in accordance with the WQ protocol. As a result, moderately lame animals were scored as not lame, which might have obscured the true number of lame animals on a few farms. Therefore, lameness might be an area of welfare concern not fully captured by the DAWIN protocol. Very few of the animal-based measures in DAWIN were behavioural, and it is therefore not possible to ascertain the extent to which behaviour was affected by the areas of concern related to the housing system.

We solicited expert opinions on the welfare consequences of each of the measures. While there is variation within experts’ opinions, the variation differs by the type of measure. For measures associated with severe pain, e.g. prolapse or tail bite, agreement tends to be higher compared to measures related to housing. However, when looking at the quartiles, the agreement is generally good. The relatively high number of experts in the current study (26) gives a good reliability of the value of the measures. The experts were chosen by three organisations: the farmers’ organisation (SEGES); the competent authority (DVFA); and the Danish Veterinary Association. While the experts therefore had different backgrounds, and possibly different interests, a corresponding study on dairy cattle, using the same set-up, found only minor differences between the different groups of experts (Otten *et al.* 2017).

For several measures identified as areas of animal welfare concern, a number of farms received the maximum score, while many scored below the threshold for acceptable welfare for these measures. This rather broad range across farms demonstrates that some were able to achieve a good welfare score while others were not. This also implies that achieving a high score for those measures was possible and therefore not unrealistic in the Danish production system. Overall, resource-based measures received lower welfare scores with higher variation both within and between farms compared to animal-based measures. This adds to the conclusion that improvements in animal welfare are to be found within the area of resources.

For all animal groups, the sample sizes used were based on the WQ protocols and therefore have the same level of representativeness as these protocols. The number of animals sampled on each farm may seem small, however, in the current project, the protocols were developed to assess welfare on a national level and the representativeness of welfare on a specific farm is therefore of less importance. Studies on the repeatability of measures subsequent to the publication of the WQ protocol have emphasised the need for choosing measures that have a high prevalence in the population of the farms (e.g. Czycholl *et al.* 2016; Friedrich *et al.* 2019a,b). The need for a high level of repeatability does however need to be weighed against the importance of the individual indicators.

What is considered to be an area of serious welfare concern overall is a combination of the values assigned by the experts (where a score of 50 or above was defined as acceptable) and the results from the farm visits. The choice of using the median of the farms visited to define areas of overall concern is however arbitrary and using different criteria will include or exclude a number of areas. The duration of a problem was not considered in the calculations due to the original aim of the DAWIN with repeated monitoring. However, a few measures of duration are included (‘Long-term crated sows’ and ‘Duration of crating – service unit’) and breeding animals are assessed throughout the production cycle adding information from each system, e.g. space in the different units.

The numbers for the proportion of farms having serious areas of animal welfare concern should be interpreted with caution as regards generalisation, since only a relatively small proportion of the farms contacted were willing to participate in the study. In addition, as stated above, the protocol was developed for use on the national level and not for welfare assessment of the individual farm. However, a few of the measures included in the DAWIN are identical to or resemble the Danish legislation. In 2012, a random sample of 299 Danish pig farms were controlled for compliance with Danish legislation (University of Copenhagen 2012) and it is therefore possible to compare the data from that study with the present to assess the representativeness of the farms visited. The two most common non-compliances were absence of available sick pens and absence of adequate rooting material. In the compliance study, 11% of the farms lacked sick pens compared to 8% in the current study. According to the legislation, soft wood but not hard, is permitted as rooting material (Ministry of Environment and Food 2021). However, no clear definition of hard versus soft wood exists. In the study assessing compliance with legislation (University of Copenhagen 2012), 24% of the farms lacked adequate rooting material. Using the same definition of rooting material 52% of the farms in the present study lacked rooting material, and if hard wood had also been considered as sufficient rooting material, 16% of the farms would still be lacking adequate material. Despite the relatively low sample size and the problems with recruitment we therefore believe that the conclusions from the sample provide a valid representation of Danish pig production.

## Animal welfare implications and conclusion

The results of this study showed several areas of animal welfare concern were observed in Danish pig herds for all four animal groups with the majority found for piglets. Inadequate space in housing systems and the type of flooring in resting areas were identified as areas of concern for all groups of pigs. Likewise, allocation of suitable rooting material or roughage was another general welfare issue. In piglets, management-induced pain was an important area of concern. Besides carpal lesions in piglets, no other clinical measures were detected as areas of concern. However, based on measure definitions and the choice of thresholds, certain clinical measures could have been underestimated and further adjustments of these measures should therefore be considered.

## Supplementary material

For supplementary material accompanying this paper visit https://doi.org/10.1017/awf.2023.37. click here to view supplementary material
