# Identifying areas of animal welfare concern in different production stages in Danish pig herds using the Danish Animal Welfare Index (DAWIN) – CORRIGENDUM

**DOI:** 10.1017/awf.2023.78

**Published:** 2023-09-04

**Authors:** Anne Marie Michelsen, Franziska Hakansson, Vibe Pedersen Lund, Marlene Katharina Kirchner, Nina Dam Otten, Matthew Denwood, Tine Rousing, Hans Houe, Björn Forkman

Upon publication of the paper the co-first authors were not equally identified.

Both Michelsen and Hakansson contributed equally to the paper.

The article has been updated to reflect this correctly.
