# Supplementary material for: Identifying areas of animal welfare concern in different production stages in Danish pig herds using the Danish Animal Welfare Index (DAWIN)
Source: Anim Welf. 2023 Jun 26;32:e47. doi: 10.1017/awf.2023.37 (PMC10936401; doi:10.1017/awf.2023.37)
Supplement: Supplementary file 1 [file S0962728623000374sup001.pdf]

Table I: DAWIN measures for gestating sows and gilts with corresponding level scores, descriptions and weightings. Level score 0 was by definition set to 100 (best possible weighting) and the remaining weightings (mean) were obtained from an expert questionnaire. Measures of variability are given for expert weightings

Principle	Measure	Level Score	Description	Weighting	Median	Q1	Q3	Min	Max
**Good feeding**	Body condition score	0	Regular	100					
		1	Very lean or very fat	33	30	20	50	10	60
	Roughage	0	Access to roughage (e.g. peat, pectin, pulp, silage, corn, whole crop, any kind of straw)	100					
		1	No access to roughage	33	38	21	44	10	55
	Feeding system (group-housed only)	0	Undisturbed feeding: *ad libitum* feeding, at least one closed feeding automat per 55 sows, one closed feeding stall per sow or evenly distributed floor feeding	100					
		1	Disturbed feeding	29	20	20	40	10	52
	Water supply	0	Sufficient: min. 2 water points when loose and 1 when crated/housed individually. When loose, at least 1 nipple per 10 sows or 1 bowl/trough per 15 sows	100					
		1	Insufficient	20	20	15	30	0	40
	Water cleanliness		Drinkers and water are without faeces or mould at the time of inspection	100					
			Drinkers and/or water are dirty with faeces and/or mould at the time of inspection	28	30	15	40	0	70
**Good housing**	Type of housing – Service unit	0	Loose-housed (group-housed or individually penned)	100					
		1	Crated	33	30	19	42	0	80
	Type of housing – Gestation unit	0	Loose-housed (group-housed)	100					
		1	Crated or individually penned	26	30	18	32	0	75
	Stocking density (group-housed only)	0	≥20% more space per sow/gilt than required by legislation	100					
		1	Compliance with legislation but less than 20% more space per sow/gilt	65	60	50	80	0	90
		2	Less space than required by legislation	29	30	20	40	0	45
	Resting area – Floorage	0	Space requirements for half recumbence (calculated using the formula: $0.033 \times$ Live weight$^{0.66}$) fulfilled	100					

Table I: (continued).

Principle	Measure	Level Score	Description	Weighting	Median	Q1	Q3	Min	Max
	(group-housed only)	1	No resting area or the space requirements for half recumbence were not fulfilled	29	28	20	40	0	70
	Resting area – Floor type*								
	Group-housed or individually penned	0	Deep bedding or soft mat in the resting area	100					
		1	Solid floor with hard mat in the resting area	57	60	50	70	0	95
		2	Solid concrete in the resting area	46	48	38	52	0	85
		3	Fully slatted or drained floor in the resting area	34	30	20	41	0	85
	Crated	0	Deep bedding or soft mat over the entire lying area	100					
		1	Mat or solid concrete floor under the front end of the sows and slatted/drained floor in the rest of the crate, or a hard rubber mat over the entire lying-area	47	50	40	60	0	95
		2	Fully slatted or drained floor over the entire area	29	30	19	40	0	85
	Duration of crating – Service unit	0	Not crated	100					
		1	≤2 weeks	43	48	26	52	0	90
		2	>2 but ≤4 weeks	34	35	23	41	0	85
		3	>4 weeks	23	20	10	30	0	80
	Crate space – Service unit	0	Suitable; crate width > depth of the sow, and crate length ≥ length of the sow + 50 cm (incl. trough)	100					
		1	Not suitable	18	18	10	20	0	50
	Cooling system	0	Cooling system present (sprinkler, cooling mats etc.)	100					
	(group-housed only)	1	No cooling system present or sows not able to avoid cooling	31	30	20	47	0	50
	Slipperiness of the floor	0	Sows do not slip on the floor	100					
		1	Sows slip ≥10 cm	31	30	20	41	5	60
	Manure on the body	0	Up to 10% of the body surface is soiled	100					
		1	>10% but ≤30% of the body surface is soiled	45	50	39	50	0	90
	(one side)	2	>30% of the body surface is soiled	30	30	20	40	0	60
	Bursitis	0	No evidence of bursitis/swelling, or bursitis/swelling ≤2 cm	100					
	(one side)	1	Bursitis/swelling >2 cm, or any eroded bursae	27	25	18	40	0	50

Table I: (continued).

Principle	Measure	Level Score	Description	Weighting	Median	Q1	Q3	Min	Max
	Panting	0	No evidence of panting	100					
		1	Evidence of panting	18	20	8	25	0	50
**Good health**	Hampered respiration	0	No evidence of hampered respiration	100					
		1	Evidence of hampered respiration	22	20	14	30	5	50
	Shoulder wounds (both sides)	0	No or small shoulder wounds; no integument changes or wounds ≤2 cm on the widest point	100					
		1	Mild shoulder wound; >2 but ≤5 cm at the widest point	22	22	14	30	0	50
		2	Severe shoulder wounds; >5 cm at the widest point	13	10	5	19	0	40
	Lameness	0	No evidence of lameness	100					
		1	Severely lame, minimum weight-bearing on the affected limb	14	15	10	20	0	30
		2	No weight-bearing on the affected limb or unable to walk	5	5	0	5	0	20
	Integument alterations	0	No evidence of lesions >5 cm in diameter (excl. scratches) or ≤10 lesions visible	100					
	(one side)	1	Lesions >5 cm in diameter (excl. scratches) or >10 lesions visible	26	28	20	36	0	50
	Vulvar lesions	0	No evidence of lesions or lesions and/or scars ≤2 cm	100					
		1	Lesions >2 cm, scars >2 cm or deformed vulva	26	28	20	36	0	40
	Prolapse	0	No evidence of prolapse (rectal, vaginal or uterine)	100					
		1	Evidence of prolapse (rectal, vaginal or uterine)	14	12	5	20	0	30
	Hernia	0	No evidence of hernia (≤5 cm in diameter)	100					
		1	Small hernia (<15 cm in diameter) with no wound or discolouration	41	42	30	50	0	100
		2	Large hernia (>15 cm diameter) or any hernia with a wound or discolouration	18	20	10	25	0	40
	Nose ring	0	Not present	100					
		1	Present	31	30	22	40	0	50
	Overgrown claws	0	Normal claw length	100					
	(both sides)	1	One or more claws are clearly overgrown	23	22	19	26	0	50

Table I: (continued).

Principle	Measure	Level Score	Description	Weighting	Median	Q1	Q3	Min	Max
	Hospital pens*	0	Hospital pens are present on-farm. Additional empty space available in pens already in use (space requirement according to Danish legislation). Floor type in resting area is deep bedding (>10 cm) or soft mat. Access to adequate, clean water supply (min. 2 water points when >5 pigs in pen)	100					
		1	No hospital pens present on-farm	12	10	9	20	0	30
		1	No empty places, or legal space requirements not fulfilled for one or more pens	17	15	10	20	0	45
		1	Solid concrete floor or hard mat	24	25	10	30	0	60
		2	Slatted or drained floor	15	15	10	20	0	35
		1	No access to water, or inadequate water supply	15	12	0	21	0	60
**Appropriate behaviour**	Stereotypies	0	No stereotypic behaviour observed	100					
		1	Stereotypic behaviour observed (e.g. sham chewing, tongue rolling, teeth grinding)	22	20	14	26	0	50
	Rooting material	0	High suitability of material (e.g. straw, roughage, branches, wood shavings, compost, peat)	100					
		1	Fair suitability of material (rope, cloth, soft wood with bark, sand)	62	70	49	80	5	95
		2	Low suitability of material (soft wood without bark)	48	52	31	60	0	80
		3	No compliance with legislation (e.g. metal, hard plastic/rubber, concrete, hard wood)	33	32	15	42	0	75
		4	No rooting material	20	15	10	26	0	50

*Integrated measure

Table II: DAWIN measures for lactating sows with corresponding level scores, descriptions and weightings. Level score 0 was by definition set to 100 (best possible weighting) and the remaining weightings (mean) were obtained from an expert questionnaire. Measures of variability are given for expert weightings

Principle	Measure	Level Score	Description	Weighting	Median	Q1	Q3	Min	Max
**Good feeding**	Body condition score	0	Regular	100					
		1	Very lean or very fat	33	30	20	50	10	60
	Roughage	0	Access to roughage (eg peat, pectin, pulp, silage, corn, whole crop, any kind of straw)	100					
		1	No access to roughage	33	38	21	44	10	55
	Feeding system (group-housed only)	0	Undisturbed feeding: *ad libitum* feeding, at least one closed feeding automat per 55 sows, one closed feeding stall per sow or evenly distributed floor feeding	100					
		1	Disturbed feeding	29	20	20	40	10	52
	Water supply	0	Functioning water point present	100					
		1	No functioning water point present	20	20	15	30	0	40
	Water cleanliness	0	Drinkers and water are without faeces or mould at the time of inspection	100					
		1	Drinkers and/or water are dirty with faeces and/or mould at the time of inspection	28	30	15	40	0	70
**Good housing**	Farrowing system	0	Lactating sows are loose-housed	100					
		1	Lactating sows housed in a SWAP-pen	68	72	50	81	0	100
		2	Lactating sows are crated	34	40	20	40	0	75
	Farrowing rails	0	No farrowing rail present	100					
		1	Farrowing rail present	25	20	16	30	0	70
	Space in farrowing system – Crate	0	Suitable; dynamic space requirements are taken into account	100					
		1	Not suitable	17	15	10	20	0	50
	Space in farrowing system – Individual pen	0	Total pen area ≥6 m^2 (incl. trough, covered creep area, etc). Sow must be able to turn easily	100					
		1	The pen area is smaller than 6 m^2 or the sow is not able to turn easily	44	45	40	50	0	75
	Space in farrowing system – Group	0	≥20% more space per sow than required by legislation	100					
		1	Compliance with legislation but less than 20% more space per sow	65	60	50	80	0	90

Table II: (continued).

Principle	Measure	Level Score	Description	Weighting	Median	Q1	Q3	Min	Max
		2	Less space than required by legislation	29	30	20	40	0	45
	Resting area – Floorage	0	Space requirements for half recumbence (calculated using the formula: 0.033 × live weight$^{0.66}$) are fulfilled	100					
	(group-housed only)	1	No resting area, or space requirements for half recumbence are not fulfilled	29	28	20	40	0	70
	Resting area – Floor type –	0	Deep bedding or soft mat over the entire resting area	100					
		1	Solid floor with hard mat in the resting area	57	60	50	70	0	95
	Group-housed or individually penned	2	Solid concrete in the resting area	46	48	38	52	0	85
		3	Fully slatted floor or drained floor over the entire area	34	30	20	41	0	85
	Crated	0	Deep bedding or soft mat over the entire lying area	100					
		1	Mat or solid concrete floor under the front end of the sows and slatted/drained floor in the rest of the crate, or a hard rubber mat over the entire lying area	47	50	40	60	0	95
		2	Fully slatted or drained floor over the entire area	29	30	19	40	0	85
	Manure on the body	0	Up to 10% of the body surface is soiled	100					
		1	>10% but ≤30% of the body surface is soiled	45	50	39	50	0	90
	(one side)	2	>30% of the body surface is soiled	30	30	20	40	0	60
	Bursitis	0	No evidence of bursitis/swelling or bursitis/swelling ≤2 cm	100					
	(one side)	1	Bursitis/swelling >2 cm, or any eroded bursae	27	25	18	40	0	50
	Long-term crated sows	0	Lactating sows not crated longer than 5 weeks	100					
		1	Lactating sows crated for longer than 5 weeks	33	32	20	46	0	60
	Panting	0	No evidence of panting	100					
		1	Evidence of panting	18	20	8	25	0	50
**Good health**	Hampered respiration	0	No evidence of hampered respiration	100					
		1	Evidence of hampered respiration	22	20	14	30	5	50
	Shoulder wounds	0	No evidence of or small shoulder wounds	100					
		1	Mild shoulder wound; >2 but ≤5 cm at the widest point	22	22	14	30	0	50
	(both sides)	2	Severe shoulder wounds; >5 cm at the widest point	13	10	5	19	0	40
	Integument alterations	0	No evidence of lesions >5 cm in diameter (excl. scratches) or ≤10 lesions visible	100					

Table II: (continued).

Principle	Measure	Level Score	Description	Weighting	Median	Q1	Q3	Min	Max
	(one side)	1	Lesions >5 cm in diameter (excl. scratches) or >10 lesions visible	26	28	20	36	0	50
	Vulvar lesions	0	No evidence of lesions or lesions and/or scars ≤2 cm	100					
		1	Lesions >2 cm, scars >2 cm or deformed vulva	26	28	20	36	0	40
	Prolapse	0	No evidence of prolapse (rectal, vaginal or uterine)	100					
		1	Evidence of prolapse (rectal, vaginal or uterine)	14	12	5	20	0	30
	Hernia	0	No evidence of hernia (≤5 cm in diameter)	100					
		1	Small hernia (<15 cm in diameter) with no wound or discolouration (>2 cm in diameter)	41	42	30	50	0	100
		2	Large hernia or any hernia with a wound or discolouration	18	20	10	25	0	40
	Nose ring	0	Not present	100					
		1	Present	31	30	22	40	0	50
	Overgrown claws	0	Normal claw length	100					
	(both sides)	1	One or more claws are clearly overgrown	23	22	19	26	0	50
	Hospital pens*	0	Hospital pens are present on-farm. Additional empty space available in pens already in use (space requirement according to Danish legislation). Floor type in resting area is deep bedding (>10 cm) or soft mat. Access to adequate, clean water supply (min. 2 water points when >5 pigs in pen).	100					
		1	No hospital pens	12	10	9	20	0	30
		1	No empty places, or legal space requirements not fulfilled for one or more pens	17	15	10	20	0	45
		1	Solid concrete floor or hard mat	24	25	10	30	0	60
		2	Slatted or drained floor	15	15	10	20	0	35
		1	No access to water, or inadequate water supply	15	12	0	21	0	60
**Appropriate behaviour**	Stereotypies	0	No stereotypic behaviour observed	100					
		1	Stereotypic behaviour observed	22	20	14	26	0	50
	Rooting material	0	High suitability of material (e.g. straw, roughage, branches, wood shavings, compost, peat)	100					
		1	Fair suitability of material (rope, cloth, soft wood with bark, sand)	62	70	49	80	5	95
		2	Low suitability of material (soft wood without bark)	48	52	31	60	0	80

Table II: (continued).

Principle	Measure	Level Score	Description	Weighting	Median	Q1	Q3	Min	Max
		3	No compliance with legislation (e.g. metal, hard plastic/rubber, concrete, hard wood)	33	32	15	42	0	75
		4	No rooting material	20	15	10	26	0	50
	Nest building	0	Possibility to perform nest-building behaviour; loose-housed and continuous access to nest material at least three days prior to farrowing and until farrowing	100					
		1	No possibility to perform nest-building behaviour; all crated sows.	23	20	14	32	0	50

*Integrated measure

Table III: DAWIN measures for piglets with corresponding level scores, descriptions and weightings. Level score 0 was by definition set to 100 (best possible weighting) and the remaining weightings (mean) were obtained from an expert questionnaire. Measures of variability are given for expert weightings

Principle	Measure	Level Score	Description	Weighting	Median	Q1	Q3	Min	Max
**Good feeding**	Age at weaning	0	≥28 days	100					
		1	21-27 days	46	50	39	50	0	90
		2	<21 days (no compliance with legislation)	20	20	10	30	0	50
	Teats per piglet	0	At least one teat per piglet	100					
		1	Less than one teat per piglet	31	30	20	45	0	60
	Water supply	0	Water point present	100					
		1	No water point present	20	20	14	26	0	40
	Water cleanliness	0	Drinkers and water are without faeces or mould at the time of inspection	100					
		1	Drinkers and/or water are dirty with faeces and/or mould at the time of inspection	32	32	25	40	0	50
**Good housing**	Stocking density	0	>0.2 m^2 per piglet	100					
		1	≥0.15 but ≤0.2 m^2 per piglet	49	50	40	60	0	90
		2	<0.15 m^2 per piglet	31	30	20	40	0	70
	Resting area – Floorage	0	All piglets have at least 0.17 m^2 each in the resting area (calculated at 7 kg using the formula: 0.047 × Live weight$^{0.66}$)	100					
		1	≥50% of piglets have 0.17 m^2 each	33	32	20	41	0	80
		2	<50% of piglets have 0.17 m^2 each	26	30	20	35	0	50
	Resting area – Floor type	0	Bedding (straw, sawdust, soft mat or similar that covers the floor occupied by the piglets)	100					
		1	Solid floor or hard mat. Sparse straw/bedding may be provided	41	40	35	50	10	80
		2	Fully drained floor	21	20	10	30	0	45
	Access to teats	0	Sufficient: Enough space on both sides of the crate based on the length of a piglet at 4 weeks of age and the depth of the sow	100					
		1	Not sufficient	36	35	30	40	0	70
	Manure on the body (one side)	0	No piglets in the litter have a soiled body surface	100					
		1	Up to 50% of piglets in the litter have a soiled body surface	37	40	30	48	0	75
		2	>50% of piglets in the litter have a soiled body surface	25	25	15	35	0	50

Table III: (continued).

Principle	Measure	Level Score	Description	Weighting	Median	Q1	Q3	Min	Max
**Good health**	Hampered respiration	0	No piglets in the litter with hampered respiration	100					
		1	One or more piglets in the litter with hampered respiration	23	25	15	30	0	50
	Lameness	0	All piglets in the litter display normal gait	100					
		1	One or more piglets in the litter display moderate lameness (difficulty in walking but still using all of its limbs) or severe lameness (minimum or no weight-bearing on the affected limb, or unable to walk)	19	20	10	30	0	50
	Lesions on the body (both sides)	0	No piglets in the litter with lesions >2 cm in diameter	100					
		1	One or more piglets in the litter with lesions >2 cm in diameter	19	20	15	28	0	40
	Carpal lesions (both sides)	0	No piglets in the litter with carpal lesions	100					
		1	Up to 50% of piglets in the litter with carpal lesions	35	30	25	45	0	80
		2	>50% of piglets in the litter with carpal lesions	20	20	15	21	0	40
	Neurological symptoms	0	No piglets in the litter with evidence of neurological symptoms	100					
		1	One or more piglets in the litter with evidence of neurological symptoms	16	15	8	22	0	40
	Diarrhoea	0	No evidence of diarrhoea present in the pen or on the piglets	100					
		1	Diarrhoea present in the pen or on the piglets	25	25	15	38	0	50
	Rectal prolapse	0	No piglets in the litter with prolapse	100					
		1	One or more piglets in the litter with prolapse	13	10	10	20	0	30
	Splay leg	0	No piglet in the litter with splay legs	100					
		1	One or more piglets in the litter with splay legs	10	10	5	12	0	30
	Castration	0	No castration performed on-farm	100					
		1	Use of analgesia and/or anaesthesia for castration	37	40	20	50	0	85
		2	No use of analgesia and/or anaesthesia for castration	14	15	0	20	0	50
	Tail docking	0	No tail docking performed on-farm	100					
		1	Use of analgesia prior to/ in connection with tail docking	40	40	20	60	0	80
		2	No use of analgesia in connection with tail docking	21	20	4	32	0	55
	Ear notching	0	No ear notching performed on-farm	100					
		1	Ear notching performed on-farm	28	30	10	40	0	80

Table IV. DAWIN measures for weaner-to-finisher pigs with corresponding level scores, descriptions and weightings. Level score 0 was by definition set to 100 (best possible weighting) and the remaining weightings (mean) were obtained from an expert questionnaire. Measures of variability are given for expert weightings

Principle	Measure	Level Score	Description	Weighting	Median	Q1	Q3	Min	Max
**Good feeding**	Body condition score	0	Regular	100					
		1	Lean	18	20	10	20	0	40
	Feeding system	0	Sufficient: *ad libitum* feeding or all pigs can eat at the same time (based on pig size)	100					
		1	Insufficient	29	30	19	40	0	60
	Water supply	0	Sufficient: min. 2 water points per pen and max. 10 pigs per nipple or 20 pigs per water bowl/ trough	100					
		1	Insufficient	20	20	15	25	0	40
	Water cleanliness	0	Drinkers and water are without faeces or mould at the time of inspection	100					
		1	Drinkers and/or water are dirty with faeces and/or mould at the time of inspection	28	30	20	40	0	45
**Good housing**	Stocking density	0	Space requirements for full recumbence (calculated using the formula: $0.047 \times$ Live weight$^{0.66}$) are fulfilled	100					
		1	Compliance with EU legislation but less than required for full recumbence	32	35	20	42	0	50
		2	Less space than required by legislation	26	22	11	30	0	95
	Resting area – Floor type	0	Deep bedding (>10 cm) or soft mat	100					
		1	Solid floor or hard mat	47	50	40	55	5	85
		2	Fully slatted or drained floor	35	40	28	40	0	75
	Resting area – Floorage	0	Space requirements for half recumbence (calculated using the formula: $0.033 \times$ Live weight$^{0.66}$) are fulfilled	100					
		1	No resting area, or space requirements for half recumbence are not fulfilled	28	30	20	35	0	75
	Cooling system	0	Cooling system present for pigs >20 kg (sprinkler, cooling mats etc.)	100					
		1	No cooling system present, or pigs not able to avoid cooling	29	30	20	40	0	60
	Slipperiness of the floor	0	Pigs do not slip on the floor	100					
		1	Pigs slip ≥10 cm	30	30	20	41	5	60

Table IV: (continued).

Principle	Measure	Level Score	Description	Weighting	Median	Q1	Q3	Min	Max
	Manure on the body	0	Up to 20% of the body surface is soiled	100					
		1	>20% but <50% of the body surface is soiled	36	40	30	42	0	75
	(one side)	2	Over 50% of the body surface is soiled	21	20	16	25	0	50
	Panting	0	No evidence of panting at pen level	100					
		1	Evidence of panting at pen level	17	20	10	22	0	40
**Good health**	Hampered respiration	0	No evidence of hampered respiration	100					
		1	Evidence of hampered respiration	20	20	12	28	0	50
	Lameness	0	No evidence of lameness	100					
		1	Severely lame, minimum weight-bearing on the affected limb	15	15	10	20	0	40
		2	No weight-bearing on the affected limb, or unable to walk	8	5	3	12	0	25
	Integument alterations	0	No evidence of lesions >5 cm in diameter (excl. scratches) or ≤10 lesions visible	100					
	(one side)	1	Lesions >5 cm in diameter (excl. scratches) or >10 lesions visible	26	25	20	35	0	49
	Tail-damage	0	No evidence of tail bites	100					
		1	Evidence of tail bites (wounds, infection, swelling, bloody appearance; total/ partial loss, healed lesions)	15	15	10	20	0	30
	Ear-damage	0	No evidence of ear-damage	100					
	(both sides)	1	Evidence of ear-damage (wounds, infection, swelling, partial or total loss of ear) or elephantiasis	17	15	10	21	0	50
	Rectal prolapse	0	No evidence of rectal prolapse	100					
		1	Evidence of rectal prolapse at pen level	9	10	5	10	0	25
	Hernia	0	No evidence of hernia (≤5 cm in diameter)	100					
		1	Pigs <30 kg: Small hernia (<10 cm in diameter) with no wound or discolouration	38	36	30	50	0	80
			Pigs >30 kg: Small hernia (<15 cm in diameter) with no wound or discolouration						
		2	Pigs < and >30 kg: Large hernia or any hernia with a wound or discolouration	15	15	6	20	0	40
	Twisted snout	0	No evidence of twisted snout	100					
		1	Evidence of twisted snout at pen level	27	25	20	40	0	50

Table IV: (continued).

Principle	Measure	Level Score	Description	Weighting	Median	Q1	Q3	Min	Max
	Neurological symptoms	0	No evidence of neurological symptoms	100					
		1	Evidence of neurological symptoms at pen level	17	15	10	28	0	30
	Liver disease	0	Pigs not affected with liver disease during the last 12 months	100					
		1	Pigs affected with liver disease during the last 12 months	25	22	11	40	0	50
	Hospital pens*	0	Hospital pens are present on-farm. Additional empty space available in pens already in use (space requirement according to Danish legislation). Floor type in resting area is deep bedding (>10 cm) or soft mat. Access to adequate, clean water supply (min. 2 water points when >5 pigs in pen)	100					
		1	No hospital pens present on-farm	14	10	5	20	0	30
		1	No additional empty spaces available in pens already in use	20	20	10	30	0	40
		1	Floor type in resting area is solid floor	32	35	20	44	0	80
		2	Floor type in resting area is slatted or drained floor	20	20	10	30	0	47
		1	No access to water, or inadequate water supply	9	5	0	19	0	30
**Appropriate behaviour**	Rooting material	0	High suitability of material (e.g. straw, roughage, branches, wood shavings, compost, peat)	100					
		1	Fair suitability of material (rope, cloth, soft wood with bark, sand)	59	65	45	80	5	95
		2	Low suitability of material (soft wood without bark)	47	50	30	60	0	85
		3	No compliance with legislation (e.g. metal, hard plastic/rubber, concrete, hard wood)	31	30	20	40	0	70
		4	No rooting material	17	15	10	25	0	50

*Integrated measure
